# Piezoelectric Biomaterials for Osteochondral Tissue Engineering: Advances, Mechanisms, and Translational Prospects

**DOI:** 10.3390/jfb17040173

**Published:** 2026-04-01

**Authors:** Hao Wang, Yunfeng Li

**Affiliations:** State Key Labqoratory of Oral Diseases, National Center for Stomatology, National Clinical Research Center for Oral Diseases, West China Hospital of Stomatology, Sichuan University, Chengdu 610041, China; 2024224035074@stu.scu.edu.cn

**Keywords:** piezoelectric biomaterials, osteochondral tissue engineering, bone regeneration, cartilage regeneration, electrical stimulation, self-powered scaffolds

## Abstract

Piezoelectric biomaterials have attracted considerable interest in osteochondral tissue engineering owing to their inherent ability to produce electrical signals in response to mechanical stimuli without external power, thereby closely mimicking the physiological electrical microenvironment required for tissue regeneration. This review comprehensively summarizes recent insights into biological piezoelectricity from the molecular to the macroscopic level, highlighting its interplay with streaming potentials and its regulatory roles in bone and cartilage regeneration. We critically analyze recent advances in major piezoelectric material systems, including ceramics, polymers, and composite scaffolds, with emphasis on their structural characteristics, bioactive performance, and suitability for tissue-specific repair. Among them, polymer-based composite and hybrid piezoelectric scaffolds appear particularly promising for the development of flexible, high-performance osteochondral repair platforms, as they offer a more favorable balance between mechanical compliance, electromechanical output, and biological adaptability. Despite encouraging preclinical findings, significant challenges remain, including biocompatibility, controlled degradation kinetics, and the precise modulation of electrical cues for specific biological contexts. To address these barriers, future research should focus on optimizing scaffold design, integrating responsive and multimodal stimulation strategies, and establishing standardized protocols for preclinical evaluation and clinical translation. Overall, piezoelectric biomaterials hold substantial potential for the development of innovative regenerative therapies for complex osteochondral defects.

## 1. Introduction

Piezoelectric materials are a unique class of functional materials characterized by their intrinsic ability to interconvert mechanical energy and electrical signals. Under mechanical deformation, these materials spontaneously generate surface charges, or conversely, they can deform under an externally applied electric field. Since the initial discovery of the piezoelectric effect by Pierre and Jacques Curie in 1880 [1,2], the underlying physical principles have been extensively studied and applied in various technological fields, including sensors, actuators, and energy harvesting systems. Structurally, piezoelectricity arises from a non-centrosymmetric lattice arrangement [3], in which mechanical stress induces asymmetrical ionic displacement and resultant polarization at the macroscopic level. More specifically, deformation causes a relative shift between the centers of positive and negative charges within the material, producing a change in dipole moment; when this polarization is not fully compensated, bound charges accumulate at the material surface or interface, thereby generating measurable electrical signals.

In recent decades, the integration of piezoelectric biomaterials into biomedical fields, particularly tissue engineering, has emerged as a promising approach due to their capability to generate stable, physiologically relevant electrical signals in response to normal physiological movements without external power sources. This unique attribute positions piezoelectric biomaterials as ideal candidates for developing self-powered, intelligent implantable devices that can actively participate in tissue regeneration by mimicking the native electrophysiological environment of living tissues. In parallel, other electrically or physically responsive material systems, such as magnetoelectric and magnetic-responsive biomaterials, have also attracted attention in biomedicine because they enable remote or non-contact stimulation. However, as their signal-generation mechanisms and material design principles differ substantially from those of piezoelectric systems, they are only briefly noted here and remain beyond the principal scope of this review.

The significance of bioelectrical signals in physiological processes such as embryogenesis, tissue homeostasis, and wound healing has been increasingly recognized, underscoring the crucial role of electrical cues in cell behavior modulation and extracellular matrix (ECM) synthesis. Biological tissues naturally exhibit endogenous piezoelectric and electrokinetic phenomena, enabling them to convert mechanical stimuli into bioelectric signals that regulate fundamental cellular activities, including proliferation, migration, differentiation, and apoptosis. Therefore, biomimetic piezoelectric scaffolds designed to replicate these inherent bioelectrical environments can provide critical guidance cues necessary for enhanced tissue regeneration.

Despite considerable advancements in osteochondral repair strategies, current clinical interventions still face significant limitations. This challenge arises in part from the marked biological differences between bone and cartilage. Bone is a highly vascularized and metabolically active tissue with substantial remodeling capacity, whereas articular cartilage is avascular, aneural, sparsely cellular, and characterized by a dense ECM and low metabolic turnover, resulting in very limited intrinsic healing potential. These differences mean that successful osteochondral regeneration requires the coordinated restoration of two structurally and functionally distinct tissues rather than the repair of a single homogeneous tissue. Traditional surgical techniques such as microfracture, autologous chondrocyte implantation, and osteochondral grafting often yield suboptimal outcomes due to issues of inadequate mechanical integration, formation of fibrocartilaginous repair tissue, donor-site morbidity, and the invasive nature of procedures. In particular, the avascular nature of cartilage severely restricts nutrient supply, endogenous cell recruitment, and matrix remodeling, thereby limiting repair efficiency. At the same time, in osteochondral regeneration, appropriate vascularization of the subchondral bone region remains essential for nutrient exchange, bone remodeling, and tissue integration, whereas excessive vascular invasion into the cartilage layer may disturb cartilage-specific regeneration and promote undesirable ossification or fibrovascular repair.

**Figure 1:**
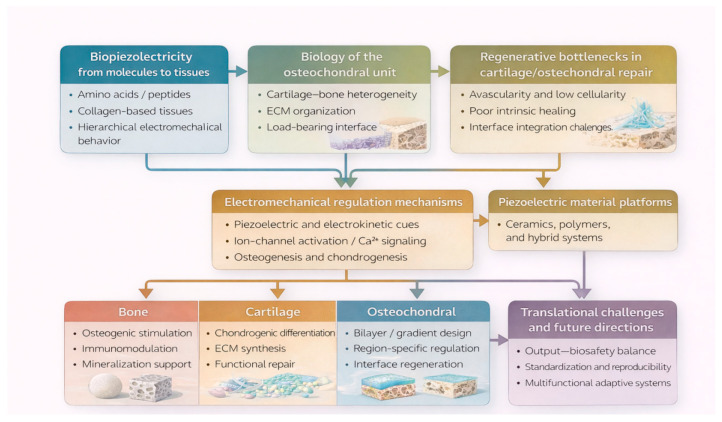
Review framework of piezoelectric biomaterials in osteochondral tissue engineering: from biological basis to translational prospects. The figure summarizes the overall logic of this review, beginning with the biological basis of biopiezoelectricity across hierarchical scales, followed by the structural and functional features of the osteochondral unit, the major regenerative bottlenecks in cartilage and osteochondral repair, the electromechanical regulatory mechanisms involved in tissue regeneration, representative material platforms for bone, cartilage, and osteochondral applications, and the key translational challenges and future directions.

Piezoelectric biomaterials present an innovative solution by providing localized electrical stimulation in situ without external power, promoting both osteogenesis and chondrogenesis. By mimicking the native electrical microenvironment, these materials effectively guide cell behavior and ECM formation through modulation of specific cellular pathways. This review systematically addresses the mechanisms and biological implications of piezoelectricity from molecular to tissue levels, explores its synergistic interaction with streaming potentials, and critically evaluates recent developments in various piezoelectric materials—ceramics, polymers, and composites. We further highlight existing challenges such as biocompatibility, controlled degradation rates, and precise electrical output regulation. Finally, future research directions are proposed to overcome current limitations and advance the clinical translation of piezoelectric biomaterials for effective osteochondral tissue regeneration. To provide a clear overview of the overall logic of this review, the major conceptual framework spanning biological basis, osteochondral biology, regenerative bottlenecks, regulatory mechanisms, material platforms, and translational prospects is summarized in Figure 1.

As shown in Figure 1, this review first examines the biological basis of biopiezoelectricity across hierarchical scales, which provides the foundation for understanding electromechanical regulation in osteochondral tissue engineering.

## 2. Biopiezoelectricity from Molecules to Tissues

Biopiezoelectricity is widely present in natural structures, spanning hierarchical systems from molecular assemblies to macroscopic organs, and has increasingly been recognized as an important biological basis for bioinspired electromechanical materials design [4,5,6]. Studies have confirmed that connective tissues such as bone and tendon, epidermal appendages such as skin and hair, and biomineralized materials such as wood and shells all exhibit measurable piezoelectric responses. Notably, this phenomenon is not confined to macroscopic tissues. At the microscopic scale, piezoelectric polarization has also been observed in biological entities such as viral capsids, protein secondary structures, amino acid crystals, and even deoxyribonucleic acid (DNA) double helices [7]. In addition, the piezoelectric behavior of neuromodulatory molecules such as tyramine and nucleotide metabolites further highlights the intrinsic relationship between bioelectric signal transmission and the mechanical microenvironment [8].

In 1940, Martin first recorded potential changes in a wool bundle, marking the beginning of experimental verification of biological piezoelectricity [9]. Subsequent studies demonstrated that mammalian hair, horns, and hooves are mainly composed of α-keratin [10,11], whose molecular chains adopt a highly ordered α-helical secondary structure stabilized by hydrogen-bond networks [11]. Further analyses revealed that the piezoelectric activity of these biological tissues originates from the periodic organization of α-helical supramolecular assemblies and the directional alignment of their internal dipole moments [12,13]. This molecular-scale symmetry breaking enables the conversion of mechanical stress into measurable polarization charges.

As the fundamental building blocks of proteins, the piezoelectric responses of amino acids are closely related to their specific spatial configurations [14,15]. When amino acids self-assemble into three-dimensional crystals, their piezoelectric behavior is closely related to crystal symmetry, and non-centrosymmetric arrangements may enable mechanoelectrical conversion comparable to that seen in piezoelectric crystalline systems [16]. Studies have also shown that the piezoelectric coefficients of amino acid crystals vary significantly with temperature, which is mainly attributed to phase-transition relaxation and energy dissipation associated with elastic vibrational modes in the lattice [17,18]. This temperature dependence is broadly consistent with the thermodynamic behavior of classical piezoelectric crystals.

As molecular assemblies formed by amino acid condensation, peptide compounds also exhibit piezoelectric activity that is strongly associated with their spatial conformation. Among them, diphenylalanine (FF), a dimer of phenylalanine residues, has become a representative model in the field of biopiezoelectric materials because of its ability to undergo supramolecular self-assembly driven by non-covalent interactions [19,20]. Studies have shown that FF molecules can spontaneously assemble into long-range ordered nanotubular structures through the synergistic effects of hydrogen-bond networks and π–π stacking [21,22,23]. Crystallographic analyses further indicate that this system exhibits non-centrosymmetric hexagonal crystal characteristics [24,25,26], providing the structural basis for its piezoelectric response.

Proteins are high-molecular-weight polymers formed by amino acids linked through peptide bonds, and their electromechanical conversion properties are closely related to molecular conformation. Studies have shown that fibrous proteins such as collagen and keratin, as well as functional proteins such as lysozyme, exhibit piezoelectric activity. In mammalian systems, collagen is a major structural protein, accounting for approximately 25–35% of total body protein [27]. Its unique supramolecular assembly plays a dominant role in the piezoelectric behavior of bone and cartilage tissues [28]. For example, type I collagen fibers in bone form a dense mineralized matrix through periodic organization. Reported piezoelectric coefficients for bone-related collagenous structures vary considerably across hierarchical and measurement scales. Values around 0.7 pC·N^−1^ have been reported for specific collagen-based measurements, whereas tissue-level measurements in human tibia have been reported in the range of 7.66–8.72 pC·N^−1^ [29,30]. Such differences likely reflect variations in sample hierarchy, orientation, hydration, and measurement mode. This electromechanical coupling property is believed to participate in the mechanotransduction processes involved in bone remodeling. In contrast, the cartilage matrix is mainly composed of type II collagen, which accounts for about 90–95% of the collagen content and resists mechanical loads through a triaxial tensile network [31,32]. Its piezoelectric response shows spatial correlation with the distribution of tissue tension. From a molecular structure–activity perspective, the characteristic triple-helical topology of collagen, in which three polypeptide chains are intertwined through hydrogen bonding [33], provides the structural basis for asymmetric charge distribution and mechanical-to-electrical energy conversion.

The piezoelectric response of collagen endows it with a unique capacity for force-to-electrical signal transduction. When tissue undergoes mechanical deformation, conformational changes in collagen molecules trigger local dipole rearrangement, thereby generating a controllable biopotential gradient. This endogenous electrical signal can be transmitted through the conductive extracellular matrix (ECM) network to adjacent cell membranes, where it may activate transmembrane signaling components such as voltage-sensitive calcium channels (VSCCs). In bone tissue, osteocytes act as the principal mechanosensory cells and form a dynamic communication network with osteoblasts and osteoclasts through piezoelectric–electrochemical coupling. Following membrane signal transduction, intracellular signaling cascades are amplified through second-messenger systems, including Ca^2+^ oscillations and the cAMP–PKA pathway, ultimately leading to the activation of nuclear transcription factors. This process regulates key physiological events such as matrix biosynthesis, cell proliferation, differentiation, and tissue repair [34]. Experimental evidence further suggests that the spatiotemporal distribution of piezoelectric signals is positively correlated with tissue regeneration efficiency.

As collagen-based tissues, the cornea and sclera also exhibit pronounced electromechanical coupling behavior, and their piezoelectric activity has been confirmed in a series of studies [35,36,37,38]. Notably, the anisotropic organization of collagen fibers in scleral tissue, which is characterized by a more disordered orientation, results in a piezoelectric coefficient of approximately 31.8 pC·N^−1^, which is markedly lower than that of corneal tissue with a more highly oriented fiber architecture. Further studies have shown that the piezoelectric responses of both the cornea and sclera are strongly dependent on hydration state. Dehydration leads to a sharp attenuation of the piezoelectric constant, which has been attributed to reconstruction of the hydrogen-bond network between collagen fibrils mediated by water molecules [38,39]. Specifically, hydration directly affects the electromechanical conversion efficiency of collagen by regulating the spatial distribution of dipole moments in polar amino acid side chains.

Prestin, a specific motor protein expressed in mammalian cochlear outer hair cells (OHCs), exhibits highly sensitive electromechanical transduction properties that do not depend on a helical conformation [40,41,42,43,44,45,46,47,48]. Real-time monitoring of OHC deformation under controlled electrical stimulation, including depolarization and hyperpolarization cycles, has confirmed its unique electromechanical coupling behavior: changes in membrane potential trigger conformational transitions of Prestin molecules, which in turn drive micrometer-scale contraction and elongation of the cell body. Further studies have shown that the intensity of the Prestin-mediated piezoelectric response is directly related to the efficiency of charge rearrangement within its transmembrane domains. Quantitative characterization using atomic force microscopy-based probes revealed that, in the approximately 50 μm-scale lateral wall membrane structure of OHCs, the equivalent piezoelectric coefficient of Prestin can reach about 20 mC·N^−1^ [49], which is several orders of magnitude higher than that of conventional piezoelectric ceramics such as PZT-5H (~600 pC·N^−1^). This extraordinary electromechanical activity is thought to arise from the synergistic coupling between biomolecular motor conformational amplification and local electric field enhancement at the cell membrane.

Lysozyme is an antibacterial protein widely present in mammalian body fluids such as tears, saliva, and milk, and its piezoelectric activity has gradually attracted attention in recent years [50,51]. In 2017, Stapleton and co-workers demonstrated through crystal engineering that lysozyme can self-assemble into two piezoelectric crystal forms: a monoclinic phase (space group P2_1_) and a tetragonal phase (space group P4_3_2_1_2) [52,53]. Quantitative characterization by piezoelectric force microscopy showed that the piezoelectric coefficient of the monoclinic crystal along the crystallographic [001] direction was 0.94 pC·N^−1^, whereas that of the tetragonal crystal increased to 3.16 pC·N^−1^, likely owing to its greater degree of lattice symmetry breaking [53]. This study provided early evidence for a structure–activity relationship between protein crystal symmetry and piezoelectric behavior, offering a new conceptual basis for the rational design of biopiezoelectric materials.

As the largest biological piezoelectric interface in the human body, skin has also been extensively investigated for its electromechanical coupling properties. In 1967, Shamos and colleagues first quantitatively characterized the piezoelectric response of skin through comparative experiments involving human, cat, and pig tissues, and proposed that this behavior was closely related to the anisotropic arrangement of collagen fibrils in the dermis. Subsequent studies in 1982 further revealed that skin, as a multilayer functional composite system composed of the epidermis, dermis, and subcutaneous tissue, exhibits marked spatial heterogeneity in piezoelectric activity [54,55,56]: the dermis, which contains abundant directionally organized type I collagen fibers, shows a typical pyroelectric–piezoelectric coupling effect, whereas the epidermis exhibits a weaker piezoelectric response mainly associated with the molecular dipole orientation of keratin [56,57]. This hierarchical electromechanical behavior provides a biophysical basis for the mechanosensory functions of skin. These biological insights have also inspired the development of piezoelectric platforms for tissue engineering and their translational exploration [58,59]. For clarity, representative piezoelectric properties of natural biological systems across molecular-to-tissue scales are summarized in Table 1. Owing to differences in structural hierarchy, testing configuration, and coefficient mode, these values should be interpreted as comparative references rather than strictly equivalent parameters.

## 3. Overview of the Biology of the Osteochondral Unit

As a multilayered composite biological structure, the function of the osteochondral unit (OCU) relies on the coordinated integration of mechanical continuity and the local biological microenvironment. It is mainly composed of two structurally and functionally distinct tissues: articular cartilage, which serves as a load-bearing and lubricating surface, and subchondral bone, which provides both mechanical support and metabolic exchange.

From a developmental perspective, the morphogenesis of the OCU originates from the spatial differentiation of limb mesenchymal cells. Between the epiphyseal ossification centers, the residual transitional mesenchymal compartment is dynamically remodeled to form the primordial joint structure [60]. As endochondral ossification progresses, mesenchymal cells in this region condense and differentiate into functionally distinct domains, ultimately giving rise to region-specific cellular subpopulations [61]. During this process, the chondrogenic layer of the outer bilayer differentiates into the epiphyseal cartilage and osteochondral junction, whereas the intermediate cavitated layer undergoes programmed cavitation to form the primordial synovial cavity [62].

The mature OCU exhibits a characteristic hierarchical organization. Articular cartilage presents a four-zone gradient architecture from the surface to the deep region, including the superficial, middle, deep, and calcified zones, each of which differs markedly in collagen fiber orientation and proteoglycan distribution [63,64]. In parallel, subchondral bone fulfills two essential functions. First, it contributes to mechanical buffering and load redistribution through its trabecular architecture. Second, it acts as a metabolic hub, with its vascularized bone matrix supporting nutrient delivery, signal exchange, and bone remodeling across the osteochondral interface [65,66,67]. Together, these features enable the OCU to maintain both biomechanical competence and tissue homeostasis under dynamic joint loading.

Compared with bone, which possesses a strong intrinsic remodeling capacity, mature articular cartilage has very limited repair potential. This difference mainly arises from their distinct microenvironmental characteristics and cellular behaviors. Bone tissue is highly vascularized, metabolically active, and able to recruit mesenchymal stem cells (MSCs) from multiple sources, including the periosteum, bone marrow, and endosteum, thereby supporting continuous remodeling and relatively effective repair [68,69]. In contrast, cartilage is characterized by low cellularity, avascularity, aneurality, and a dense extracellular matrix (ECM), all of which restrict endogenous repair. These features not only limit nutrient transport and progenitor cell recruitment but also create physical barriers to exogenous cell infiltration and matrix remodeling.

It is also important to note that the cartilage ECM itself is metabolically heterogeneous. Glycosaminoglycan (GAG) components retain a certain renewal capacity [70], whereas the type II collagen (Col II) network exhibits an extremely long metabolic half-life [71]. This imbalance in matrix turnover makes it difficult for mature cartilage to reconstruct an intact Col II framework after injury. Because the spatial organization and three-dimensional topology of Col II are fundamental to the tensile strength and load-bearing behavior of cartilage, failure to restore this network often leads to structurally and functionally inferior repair tissue [72].

Successful osteochondral regeneration therefore requires more than isolated cartilage repair. It requires coordinated reconstruction of cartilage, calcified cartilage, and subchondral bone, together with proper regulation of interfacial mechanics, matrix organization, and vascular support. In particular, although mature articular cartilage itself is avascular, appropriate vascularization in the subchondral bone region remains essential for nutrient supply, metabolic communication, and structural integration. However, excessive vascular invasion into the cartilage layer may disrupt cartilage-specific regeneration and contribute to fibrovascular repair or ectopic ossification. These biological complexities are a major reason why osteochondral defect repair remains a persistent clinical challenge.

## 4. Regenerative Bottlenecks in Cartilage Repair and Electrical Stimulation-Based Intervention Strategies

Articular cartilage injury is a chronic and progressive degenerative condition that represents a major cause of disability across different age groups and imposes a substantial public health burden [73,74]. It has been estimated that the number of affected adults in the United States has exceeded 50 million and may rise to more than 67 million by 2030 [75]. Owing to its avascular and aneural nature, articular cartilage has very limited spontaneous repair capacity when subjected to cumulative microdamage under long-term mechanical loading [76]. As a result, even minor injuries may progressively develop into irreversible structural defects if timely and effective intervention is not achieved. This impaired regenerative capacity is closely associated with several intrinsic biological constraints, including restricted recruitment of reparative cells and signaling factors, limited metabolic exchange, and the inability to rapidly restore tissue-specific extracellular matrix (ECM) architecture.

In addition to its poor intrinsic healing capacity, cartilage exhibits pronounced zonal heterogeneity, extending from the superficial collagen-rich layer to the deep calcified zone. This gradient organization creates several major challenges for successful repair. First, repair materials must accommodate the distinct shear, tensile, and compressive demands of different cartilage layers. Second, regenerated tissue must achieve seamless integration with surrounding host cartilage to restore structural continuity and effective force transmission. Third, long-term repair depends on the establishment of a biologically functional microenvironment capable of supporting nutrient exchange, cellular activity, and matrix homeostasis. These challenges help explain why currently available clinical strategies still yield limited long-term outcomes.

Commonly used clinical approaches for cartilage repair include microfracture (MF) stimulation [77], autologous chondrocyte implantation (ACI) [78], and osteochondral autograft or allograft transplantation (OAT) [79,80]. Although these techniques have shown clinical utility in selected cases, their outcomes remain constrained by multiple limitations. For example, the repair tissue generated after MF is predominantly fibrocartilage, often accounting for more than 70% of the regenerated tissue, and is enriched in collagen type I rather than the collagen type II-dominant structure of native hyaline cartilage [81]. Consequently, its mechanical performance is inferior and it is more prone to mid- to long-term degeneration. Transplantation-based strategies are additionally limited by donor scarcity, donor-site morbidity in autografts, and possible immune rejection in allografts. Furthermore, a mechanical mismatch between graft and host tissue may result in fibrous interfacial tissue formation, thereby compromising structural integration and efficient mechanical signal transmission [82]. The invasive nature of these procedures also introduces surgical risks, including infection and the need for secondary intervention [83].

Tissue engineering has therefore emerged as a promising strategy for cartilage regeneration, particularly through the design of functional scaffolds and the modulation of the repair microenvironment [55,84]. Effective cartilage repair requires the coordinated integration of chemical and physical cues. In addition to biochemical signals such as growth factors and cytokines, physical stimulation—including mechanical and electrical cues—plays an important role in regulating cell proliferation, lineage-specific differentiation, and ECM synthesis [85].

Among these approaches, electrical stimulation (ES) has been widely recognized as an effective physical strategy for promoting chondrocyte activity and extracellular matrix production in vitro and in vivo [86,87,88,89]. Endogenous bioelectrical cues are believed to participate in the regulation of cartilage-related cell behavior and tissue organization, which also supports the rationale for applying electrical stimulation in cartilage regeneration [86,87]. Mechanistically, electrical fields can regulate ion-channel activity, membrane polarization, and downstream signaling pathways, thereby influencing cell behavior and tissue organization. In parallel, the biphasic structure of cartilage, consisting of a collagen–proteoglycan matrix and interstitial fluid, makes it particularly responsive to electrical and electrokinetic cues [86,90].

Cartilage also exhibits a depth-dependent electromechanical gradient. From the superficial to the deep zone, electrical resistivity increases from approximately 1.2 Ω·m to 3.8 Ω·m, while the elastic modulus rises from about 0.5 MPa to 15 MPa and permeability decreases by nearly two orders of magnitude [91]. Together, these spatial variations establish a structurally coupled mechano-electrical microenvironment that is highly relevant to cartilage homeostasis and repair.

Exogenous ES can modulate cartilage metabolism partly through the regulation of extracellular calcium dynamics. For example, low-frequency pulsed extracellular Ca^2+^ oscillations (0.1–1 Hz) activate the CaM/CREB signaling axis and promote the expression of collagen type II and aggrecan [92], whereas sustained high extracellular Ca^2+^ levels may inhibit SOX9 transcription and impair chondrogenesis [93]. Through this multistep signaling cascade, ES provides a potentially non-invasive and relatively precise means of regulating cartilage-related anabolic and catabolic processes.

**Figure 2:**
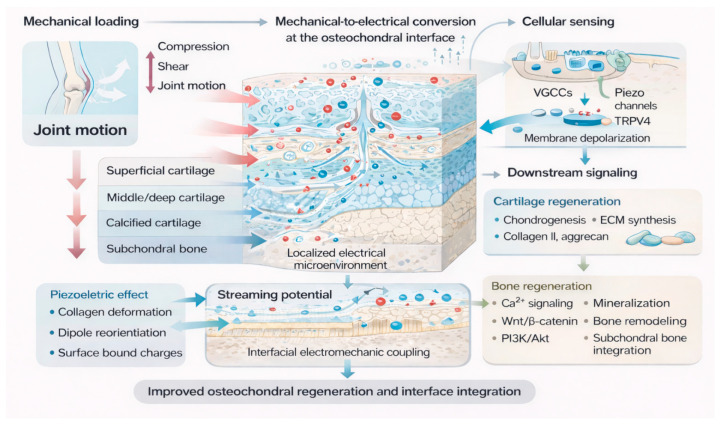
Schematic illustration of mechanical-to-electrical signal conversion at the osteochondral interface and its biological consequences. Physiological joint loading, including compression, shear, and motion, induces extracellular matrix deformation and interstitial fluid movement within the osteochondral unit. These mechanical stimuli generate localized electrical cues through piezoelectric and electrokinetic mechanisms, including collagen-associated polarization, bound charge formation, and streaming potentials. The resulting electrical microenvironment is sensed by cells through ion channels such as VGCCs, Piezo channels, and TRPV4, leading to membrane depolarization, Ca^2+^ influx, and activation of downstream signaling pathways. These events subsequently regulate chondrogenesis, osteogenesis, extracellular matrix synthesis, mineralization, and osteochondral interface integration.

Although animal studies have shown that ES can promote chondrocyte proliferation and enhance the synthesis of collagen type II, proteoglycans, and glycosaminoglycans (GAGs) [87,88,89], most conventional ES systems rely on direct-current contact or capacitive coupling devices, which face several practical limitations, including infection risk, patient discomfort, and complications associated with device implantation [94]. In this context, piezoelectric biomaterials have attracted increasing interest because of their self-powered properties, which eliminate the need for continuous external energy input [95,96]. By converting physiological joint motion into localized electrical signals, these materials can mimic the native electrical microenvironment of cartilage and support the development of self-powered regenerative microenvironments. Therefore, piezoelectric strategies offer a structurally simple, physiologically responsive, and potentially translational approach for implementing electrical stimulation in osteochondral tissue engineering [97]. As illustrated in Figure 2, physiological mechanical loading can be converted into localized electrical cues at the osteochondral interface through piezoelectric and electrokinetic mechanisms, which subsequently regulate ion-channel activity, intracellular signaling, and tissue-specific regenerative responses.

## 5. Advances in the Application and Mechanisms of Piezoelectric Functional Materials in Bone Tissue Engineering

With the development of biomaterials science, traditional inert bone repair materials are gradually evolving into functional systems with bioactivity and signal-regulating capability. Among them, piezoelectric materials have attracted widespread attention because they can spontaneously generate electrical signals under mechanical stimulation and thereby provide biomimetic electromechanical cues without continuous external power input. By partially mimicking the endogenous bioelectrical environment of bone tissue, these materials can participate in the regulation of cell behavior, matrix deposition, and tissue regeneration. In recent years, a variety of piezoelectric ceramics, polymers, and composite systems have been developed for bone tissue engineering, progressively establishing a mechanistic framework linking piezoelectric stimulation to cellular responses and tissue repair. In general, ceramic materials provide relatively strong electromechanical output, polymeric materials offer superior flexibility and tissue adaptability, and hybrid or composite systems are increasingly recognized as promising platforms because they can better integrate electrical functionality, mechanical compliance, and biological compatibility. The following sections summarize the major regulatory mechanisms and representative advances in the application of piezoelectric materials in bone tissue engineering, with particular emphasis on how piezoelectric signals modulate osteogenesis.

### 5.1. Osteogenic Regulatory Mechanisms Mediated by Piezoelectric Signals

Recent studies have increasingly focused on the molecular mechanisms by which piezoelectric stimulation regulates bone remodeling and osteogenic differentiation. Two major aspects have been emphasized. First, piezoelectric stimulation can modulate cellular function through electrophysiological mechanisms. Exogenous electrical cues may activate voltage-gated sodium and calcium channels (VGSCs/VGCCs), leading to membrane depolarization, oscillatory bioelectrical activity, and altered intracellular ion homeostasis. Through regulation of Na^+^/K^+^-ATPase activity and Ca^2+^-dependent signaling, these processes influence cellular metabolism and promote osteogenic phenotype expression [98].

Second, characteristic endogenous bioelectrical templates exist in the bone injury microenvironment. Following tissue disruption, resting potential gradients are altered, and studies have shown that the potential gradient in bone defect regions ranges from approximately −52 to −87 mV, compared with −10 to −30 mV in healthy bone tissue [99]. These electrochemical differences are considered important endogenous cues in bone regeneration. Accordingly, biomimetic material design for functional bone repair should ideally address several aspects, including reconstruction of appropriate interfacial electrical environments, regulation of ion-associated signaling dynamics, and synergistic activation of osteogenesis-related pathways such as Wnt/β-catenin and RANKL/OPG. Through these mechanisms, piezoelectric microstimulation may help coordinate osteoblast–osteoclast coupling and support bone regeneration.

Based on these mechanistic insights, recent research has further explored a range of piezoelectric ceramics, polymers, and composite systems for bone tissue engineering. The following sections summarize representative developments in these material categories.

### 5.2. Research Progress of Piezoelectric Ceramic Materials in Bone Repair

#### 5.2.1. Self-Powered Piezoelectric Ceramics and Electrets

Achieving bone repair stimulation without external power input represents an important step toward the clinical translation of piezoelectric materials. Compared with conventional electrically powered stimulation systems, self-powered piezoelectric systems have attracted increasing attention in bone tissue engineering because of their inherent energy-harvesting capability, relatively simple structure, and potential in vivo applicability. These systems can convert physiological loading, muscle contraction, or microenvironmental perturbation into electrical signals through electromechanical coupling, thereby activating osteogenesis-related pathways and helping establish a biomimetic electrical microenvironment.

For example, Zhang and co-workers developed a self-driven pulsed direct-current stimulation system by integrating a shape-memory-driven arch-shaped piezoelectric nanogenerator (sm-PENG) with an orthopedic external fixation splint [100]. This system exhibited energy self-sufficiency through cyclic deformation-induced piezoelectric output, producing peak currents up to 20 μA and pulse waveforms resembling physiological electrical stimulation. Biologically, it enhanced MC3T3-E1 cell viability during long-term culture, increased alkaline phosphatase (ALP) activity, upregulated osteogenic genes such as Runx2 and Osterix, and promoted matrix mineralization. This type of integrated design illustrates the potential of coupling piezoelectric materials with force-generating systems to construct self-powered bone stimulation platforms.

At the single-material level, Yu et al. developed a porous electret nanofiber mat (HCBG) composed of polycaprolactone (PCL) and liquid paraffin, which generated stable electrical output driven by host muscle movement and thereby enabled in vivo self-powered osteogenic stimulation [101]. Under optimized conditions, HCBG produced voltages up to 40 V and current densities of 0.98 μA/cm^2^ while maintaining more than 80% of its electrical performance after 21 days of PBS immersion. In vitro, the material significantly enhanced BMSC ALP activity, calcium nodule formation, and osteogenic gene expression. In a rat femoral defect model, it supported near-complete bone regeneration within eight weeks. Mechanistically, this effect was associated with intracellular Ca^2+^ elevation and activation of the CaM/CaN/NFAT pathway. In a related approach, Das et al. also reported a biodegradable nanofibrous scaffold that functioned as a remotely controlled, self-powering electrical stimulator for bone regeneration, further supporting the feasibility of integrating degradable scaffold design with self-powered osteogenic stimulation [102].

In another study, Zhang et al. proposed a strategy to promote bone regeneration by regulating the polarity of GaN/AlGaN heterostructures and utilizing their intrinsic electric fields [103]. GaN/AlGaN nanofilms with negative-polarity and positive-polarity surfaces were fabricated, and the negatively polarized surfaces showed superior osteogenic performance. In vivo, the negatively polarized group exhibited higher new bone volume and bone–implant contact than the positively polarized group and SiC controls. Transcriptomic analysis further suggested that this effect involved activation of TGF-β, PI3K-Akt, focal adhesion, and ECM–receptor interaction pathways, with BMP6 emerging as a possible electrically responsive osteogenic regulator.

Another representative example is the sandwich-structured SiO_2_/PDMS electret membrane (E-S/P) designed by Qiao et al. [104]. After SiO_2_ nanoparticle incorporation and polarization treatment, this membrane exhibited a stable negative surface potential capable of simulating a physiologically relevant electrical environment. In vitro, the E-S/P membrane enhanced BMSC adhesion, migration, and osteogenic differentiation, while in a rat cranial defect model, it significantly promoted new bone formation and integration with host tissue. Collectively, these studies highlight how the coordinated interplay among material properties, mechanical activation, and cellular signaling can support the establishment of a regenerative electrical microenvironment for bone repair.

#### 5.2.2. Lead-Based Ceramic Benchmarks and the Shift Toward Lead-Free Alternatives

Lead-based piezoelectric ceramics, particularly PZT and PMN-PZT, have long served as benchmark materials because of their high piezoelectric coefficients and reliable electromechanical performance. Their d_33_ values can exceed 500 pC·N^−1^, making them highly attractive for sensing, actuation, and stimulation-related applications [52,105]. In bone-related technologies, these materials have been explored in systems such as wearable bone density monitoring and ultrasound-associated stimulation devices, where strong and stable electrical output is advantageous. Their use has helped establish the concept that piezoelectric stimulation can actively regulate bone-related biological responses.

However, despite their excellent electrical properties, the application of lead-based ceramics in bone tissue engineering remains severely constrained by several intrinsic drawbacks. The potential release of lead ions raises major biosafety concerns, particularly in long-term implantation scenarios. In addition, these ceramics are generally non-degradable and mechanically brittle, which compromises their compatibility with regenerating tissue and limits their ability to integrate with dynamic biological environments. These limitations collectively restrict their translational potential in regenerative medicine, even though they continue to serve as important performance benchmarks.

Accordingly, current research has increasingly shifted toward lead-free piezoelectric ceramics and biologically safer material systems. Nevertheless, lead-free ceramics should not yet be regarded as complete one-to-one substitutes for lead-based benchmark materials such as PZT. Although representative lead-free systems such as KNN and BaTiO_3_ have shown encouraging biocompatibility and osteogenic activity, they still generally fall short of lead-based ceramics in terms of overall electromechanical output, processing reproducibility, and long-term functional reliability. Therefore, they are more appropriately considered promising alternatives whose clinical value will depend on further optimization of material performance, structural stability, and biological integration. Representative lead-free ceramic systems are discussed in the following section.

### 5.3. Representative Lead-Free Piezoelectric Ceramics for Bone Repair

Among the currently investigated lead-free piezoelectric ceramics, potassium sodium niobate (KNN), magnesium silicate (MgSiO_3_), and barium titanate (BaTiO_3_) are representative systems that have attracted particular attention in bone tissue engineering because of their distinct combinations of piezoelectric activity, biological response, and translational potential.

Potassium sodium niobate (KNN)-based ceramics are widely regarded as promising lead-free candidates for biomedical piezoelectric applications because of their relatively high piezoelectric activity, favorable energy-conversion efficiency, and good thermal stability. Representative parameters include a piezoelectric coefficient of d_33_ ≈ 260 pC·N^−1^, electromechanical coupling coefficients of k_33_ = 53% and k_p_ = 48%, and a Curie temperature of approximately 420 °C [106,107,108]. Their lower density compared with PZT is also advantageous from a materials-design perspective [107,109]. However, despite these attractive characteristics, the biomedical translation of KNN remains challenging because of concerns related to brittle fracture behavior, uncertain long-term biocompatibility, hydrolytic instability in physiological environments, and demanding fabrication conditions [109]. For example, Zheng et al. developed a Zn-doped KNN/PLGA composite scaffold that generated approximately 40 mV under ultrasonic excitation and exhibited antibacterial and osteogenic activities. Nevertheless, the functional utilization of its piezoelectric potential remained limited, and issues such as ceramic agglomeration, weak interfacial bonding, and insufficient functional integration were not fully resolved [110]. These findings indicate that, although KNN is a promising lead-free system, further optimization is still required before it can approach the overall practical utility of lead-based ceramic benchmarks.

Magnesium silicate (MgSiO_3_), a lead-free piezoelectric bioceramic with an asymmetric tetragonal crystal structure, has also attracted attention as a potential bio-piezoelectric bone substitute because of its biodegradability and favorable biocompatibility [99,111,112,113]. During degradation, MgSiO_3_ releases Mg^2+^ and Si-related ionic species, which may promote mesenchymal stem cell osteogenic differentiation and support bone formation [113]. However, compared with conventional piezoelectric ceramics such as BaTiO_3_, MgSiO_3_ exhibits weaker piezoelectric output and therefore provides less intense electrical stimulation. In addition, its degradation behavior is not yet easy to match precisely with the rate of new bone formation. Although Mg^2+^ release may support angiogenesis by enhancing endothelial cell migration, excessive ion release can interfere with osteoblast mineralization and disrupt local ionic homeostasis, thereby adversely affecting the bone regeneration microenvironment. Potential strategies to better regulate the degradation behavior of MgSiO_3_-based systems include compositional modification or ion doping, surface coating, control of porosity and microstructure, and incorporation into polymer–ceramic composite scaffolds. These approaches may help better coordinate scaffold resorption, ionic release, mechanical integrity, and piezoelectric function with the dynamic process of bone repair. Therefore, despite its biological advantages, MgSiO_3_ still requires further improvement in both piezoelectric performance and degradation control before it can be considered a mature piezoelectric bone substitute.

Barium titanate (BaTiO_3_), a typical perovskite ferroelectric ceramic, is among the most extensively studied lead-free piezoelectric biomaterials. It possesses relatively strong piezoelectric performance, good biocompatibility, and notable osteoinductive activity [114]. BaTiO_3_ can direct mesenchymal stem cells toward osteogenic differentiation [115], and as a nanoscale filler, it can also improve the mechanical properties of polymeric matrices such as polylactic acid [116], highlighting its value not only as a standalone ceramic but also as a functional component in composite systems. Below its Curie temperature, BaTiO_3_ adopts a tetragonal structure, and its piezoelectric behavior arises from lattice-level displacement polarization associated with Ti^4+^ and O^2−^ ions. However, although BaTiO_3_ is one of the most promising lead-free alternatives, it still does not universally match PZT in high-end electromechanical performance across all application contexts. Its translational significance may therefore lie less in directly replacing PZT and more in serving as a biologically safer piezoelectric phase in multifunctional biomedical systems.

For instance, Wu et al. developed a polarized BaTiO_3_-coated titanium alloy scaffold with a piezoelectric constant of up to 3.2 pC·N^−1^, approaching the range reported for natural bone [117]. Under physiological loading or low-intensity pulsed ultrasound, this scaffold continuously generated microcurrents and promoted macrophage polarization toward the anti-inflammatory M2 phenotype, with subsequent IL-10 and TGF-β1 secretion supporting osteogenic differentiation. In a large-animal model, the BaTiO_3_-coated scaffold achieved significantly greater new bone formation than the titanium control group, demonstrating substantial osteoimmunomodulatory potential.

Wu et al. also reported a piezoelectric hydrogel scaffold (CG/PHA/5%PBT) integrating immunomodulatory, angiogenic, and osteogenic functions [118]. Under microforce stimulation, this scaffold generated sustained electrical output and promoted macrophage M1-to-M2 polarization, upregulated pro-regenerative cytokines, enhanced endothelial lumen formation, and increased the expression of osteogenic markers such as ALP, Runx2, Col1, and OCN. In a critical-sized cranial defect model, the scaffold supported substantial new bone formation and favorable tissue integration. Together, these findings suggest that the most meaningful translational role of BaTiO_3_ may be as part of hybrid or multifunctional platforms that balance biological safety, electrical activity, and structural adaptability for bone regeneration.

### 5.4. Advantages and Tissue Compatibility of Piezoelectric Polymer Materials

Compared with piezoelectric ceramics, piezoelectric polymers generally exhibit lower piezoelectric coefficients, but they possess several distinctive advantages that make them highly attractive for bone tissue engineering. In particular, their superior flexibility, compliance, processability, and structural tunability allow them to better adapt to complex biological environments and dynamic tissue interfaces. Through fabrication techniques such as electrospinning, spin coating, and templating, piezoelectric polymers can be processed into a wide range of architectures, including nanofibers, films, microspheres, and hydrogels, thereby enabling more precise mimicry of native extracellular matrix microstructures and tissue-scale mechanical behavior [119].

Among these materials, polyvinylidene fluoride (PVDF) and its copolymer poly(vinylidene fluoride-trifluoroethylene) [P(VDF-TrFE)] are the most extensively studied piezoelectric polymers because of their relatively high β-phase content and tunable electromechanical performance [120]. The piezoelectricity of PVDF originates from the oriented alignment of C–F dipoles within its β-phase crystalline structure, which exhibits the most favorable piezoelectric properties among its crystalline phases [121]. Although the piezoelectric coefficients of these polymers remain lower than those of most ceramic counterparts, their mechanical softness and adaptability are advantageous for generating physiologically compatible electrical stimulation under dynamic loading conditions.

A variety of strategies have been developed to further enhance the piezoelectric output of PVDF-based materials. These include high-voltage poling, mechanical stretching, and the incorporation of conductive or functional nanofillers, all of which can promote dipole alignment and increase β-phase content [122,123]. Electrospinning is one of the most widely used techniques for fabricating piezoelectric PVDF nanofibers, because the combined effects of electric-field stretching and mechanical drawing can substantially enrich the β-phase while producing ECM-like fibrous structures favorable for cell attachment and growth [58,119]. In addition, permanently hydrophilic electrospun PVDF nanofibrous scaffolds have been shown to promote osteoblast activity through unaided electromechanical stimulation, further highlighting the potential of surface-engineered PVDF platforms for bone regeneration [124]. For example, Szewczyk et al. regulated the surface potential of electrospun PVDF scaffolds by controlling the polarity of the poling voltage, and showed that negatively polarized PVDF scaffolds with surface potentials close to typical cell membrane potentials significantly promoted MG63 cell adhesion, spreading, proliferation, collagen fiber formation, and early mineralized nodule generation [5]. These findings suggest that piezoelectric polymer surfaces can actively modulate the osteogenic microenvironment at the cell-material interface.

In addition to PVDF-based systems, biodegradable piezoelectric polymers such as poly(L-lactic acid) (PLLA) and polyhydroxyalkanoates (PHA) have attracted increasing attention because they combine intrinsic piezoelectricity with degradability and favorable biocompatibility [119,125,126]. Their degradation behavior can be tuned to some extent, which offers additional flexibility for scaffold design in regenerative applications. Compared with ceramic materials, piezoelectric polymers therefore provide clear advantages in compliance, interfacial adaptability, and structural processability. However, their relatively weak electrical output often limits their effectiveness as standalone high-performance stimulatory materials.

For this reason, hybrid and composite piezoelectric systems are increasingly considered the most promising direction for future development. By integrating polymer matrices with piezoelectric ceramic phases or other functional components, these systems can combine the flexibility, degradability, and tissue compatibility of polymers with the stronger electromechanical activity of inorganic materials. Such a design strategy is particularly relevant for bone tissue engineering, where successful regeneration requires not only sufficient electrical stimulation but also appropriate mechanical matching, structural stability, and long-term biological integration. Therefore, polymer-based hybrid piezoelectric scaffolds may represent a more realistic and translationally attractive route for the development of clinically applicable bone repair materials.

Taken together, ceramic, polymeric, and hybrid piezoelectric materials each offer distinct advantages for bone regeneration, and their rational integration may provide the most effective route toward functionally optimized bone repair platforms [58,121].

## 6. Advances in the Application of Piezoelectric Materials in Cartilage Tissue Engineering

### 6.1. Mechanisms by Which Piezoelectric Materials Regulate Chondrocyte Behavior

Electrical stimulation (ES) can effectively induce mesenchymal stem cells (MSCs) to undergo chondrogenic differentiation through multiple signaling pathways. These mechanisms involve Ca^2+^ influx mediated by voltage-gated calcium channels (VGCCs) and mechanosensitive ion channels such as Piezo and TRPV4, which activate the CaM/CaN–NFAT signaling axis and thereby promote the expression of cartilage-related genes, including SOX9, COL2A1, and ACAN. In parallel, the cAMP/PKA and JNK/CREB/STAT3 pathways can synergistically enhance the TGF-β/BMP signaling cascade, further supporting cell differentiation and extracellular matrix (ECM) synthesis [127,128]. In addition, engineered electroactive culture systems and electrically conductive scaffold platforms have provided practical support for the controlled delivery of electrical cues in tissue-engineering settings, further highlighting the importance of structural context in electrical stimulation-based regeneration [57,129]. Lehmenkötter et al. demonstrated that applying sinusoidal electrical stimulation (2.5 V, 8 Hz) to MSCs significantly increased cell viability and ACAN expression, confirming that ES can modulate stem cell fate toward a chondrogenic phenotype [127]. In addition, previous studies have also suggested that electrical stimulation applied within three-dimensional scaffold environments can help preserve chondrocyte-like morphology and enhance extracellular matrix production, indicating that the structural context of stimulation is important for cartilage regeneration [127,128,129,130]. Hiraoka et al. further showed that the combination of moderate electrical stimulation (MES) with heat treatment inhibited HSP70 degradation, thereby enhancing its stability and indirectly promoting the expression of type II collagen and proteoglycans, which suggests a potential protective stress-response mechanism associated with ES [131].

Given that piezoelectric signals can induce chondrogenic phenotypes by regulating ion channels and downstream signaling pathways, recent research has increasingly focused on developing piezoelectric material systems capable of providing continuous and localized electrical stimulation in cartilage-related tissue environments. Representative platforms for cartilage regeneration are discussed in the following sections.

### 6.2. Representative Piezoelectric Platforms for Cartilage Regeneration

#### 6.2.1. Scaffold-Based Piezoelectric Systems

Liu et al. developed a biodegradable osteochondral scaffold system with dual piezoelectric and conductive functionalities by combining decellularized cartilage extracellular matrix (dECM) with polyaniline-modified gelatin (Gel-PC) in a heterogeneous bilayer structure designed to mimic the bidirectional inductive functions of the native osteochondral interface [132]. Under mechanical stimulation generated by joint movement, the scaffold produced self-driven electrical outputs of approximately 20 mV and established a stable spatial electrical potential gradient. In this system, the positively charged region promoted BMSC differentiation toward chondrogenic phenotypes, whereas the negatively charged region favored osteogenic differentiation. In vitro experiments showed significant upregulation of cartilage-related markers such as SOX9 and COL2A1, as well as bone-related markers including Runx2 and ALP. Transcriptomic analysis further revealed enrichment of FoxO and TGF-β signaling pathways in a tissue-specific manner. In a porcine model, the scaffold supported continuous osteochondral interface regeneration over 12 weeks, with repaired tissue showing mechanical properties and collagen fiber organization approaching those of native tissue. This study highlights the potential of spatially organized electromechanical scaffolds for functional osteochondral regeneration.

Xie et al. developed a three-dimensional composite scaffold integrating piezoelectric functionality with biofactor synergy to enhance articular cartilage regeneration [133]. The scaffold consisted of a polylactic acid (PLLA)/barium titanate (BaTiO_3_) composite backbone, with a collagen/FGF-18 coating applied to the bottom layer to provide both electromechanical stimulation and controlled biofactor release. Under mechanical loading, the scaffold generated piezoelectric charges that mimicked the native electrical microenvironment of cartilage, while sustained FGF-18 release promoted ECM synthesis and cell migration. In vitro, the scaffold enhanced chondrocyte proliferation, collagen secretion, and type II collagen expression. In a rabbit joint defect model, it supported high-quality cartilage regeneration and tissue integration within 8 weeks, illustrating the value of combining piezoelectric stimulation with growth factor-mediated repair.

In another representative study, Jacob et al. fabricated a nanohybrid piezoelectric scaffold based on poly(3-hydroxybutyrate-co-3-hydroxyvalerate) (PHBV) and BaTiO_3_ [134]. The scaffold, produced by electrospinning, exhibited a highly porous ECM-like architecture with a large surface area. Incorporation of 20% BaTiO_3_ increased the piezoelectric coefficient to approximately 1.4 pC·N^−1^, which is close to the reported range of natural cartilage, and significantly improved the mechanical properties of the scaffold. After polarization treatment, the material showed enhanced bioactivity and significantly promoted adhesion, proliferation, and type II collagen expression in human mesenchymal stem cell-derived chondrocytes without requiring an external power supply. In particular, the PB20-BT20 group exhibited marked upregulation of COL2A1 and favorable cell morphology, suggesting that nanohybrid piezoelectric scaffolds can support cartilage regeneration by reproducing aspects of the physiological electrical microenvironment in a self-powered manner.

Taken together, scaffold-based piezoelectric systems have evolved from simple electrically responsive constructs into multifunctional regenerative platforms that integrate electromechanical stimulation, matrix mimicry, interface guidance, and bioactive regulation. These features make them especially attractive for cartilage and osteochondral regeneration, where both structural and microenvironmental complexity must be considered.

#### 6.2.2. Injectable Piezoelectric Hydrogel Systems

In recent years, piezoelectric hydrogels have attracted considerable interest in cartilage tissue engineering because of their injectability, soft-tissue adaptability, and favorable biocompatibility. Vinikoor et al. developed a piezoelectric composite hydrogel composed of short-fiber poly(L-lactic acid) (PLLA) embedded in a collagen matrix, which exhibited biodegradability and ultrasound responsiveness [135]. Upon ultrasound activation, the hydrogel generated stable weak electrical signals that markedly promoted the migration and chondrogenic differentiation of adipose-derived mesenchymal stem cells (ADSCs), while also inducing endogenous TGF-β1 secretion. This process upregulated the expression of cartilage-specific genes such as SOX9, COL2A1, and ACAN. In a rabbit osteochondral defect model, the hydrogel facilitated the regeneration of hyaline cartilage-like tissue within 8 weeks, accompanied by subchondral bone remodeling and favorable mechanical restoration. Repair outcomes were significantly better than those of the non-activated control group. This study demonstrates the potential of injectable piezoelectric hydrogels as minimally invasive platforms that enable physical stimulation-triggered cartilage repair without the need for exogenous growth factors.

#### 6.2.3. Mechano-Electric Responsive Microsphere Systems

Han et al. developed an injectable mechano-electric transduction hydrogel microsphere system (Piezo@CR MPs) that integrates piezoelectric nanoparticles with stem cell recruitment functionality, thereby enabling targeted stem cell aggregation and sustained electrical stimulation in a synergistic manner [136]. The microspheres consisted of a methacrylated hyaluronic acid (HAMA) matrix doped with barium titanate nanoparticles and surface-modified with dopamine and a stem cell-recruiting peptide (E7 sequence). Under ultrasound activation, the system continuously generated stable electrical signals with a maximum voltage of 451 mV while simultaneously releasing the peptide to induce directional migration and local enrichment of bone marrow-derived mesenchymal stem cells (BMSCs). In vitro, the platform significantly promoted BMSC chondrogenic differentiation, as indicated by increased expression of ACAN, COL2A1, and SOX9, together with enhanced glycosaminoglycan synthesis. Mechanistically, Piezo@CR MPs induced intracellular Ca^2+^ influx and activated the p38 MAPK signaling pathway, thereby driving chondrogenic differentiation. In rabbit osteochondral defect and rat osteoarthritis models, this system significantly improved cartilage quality and functional recovery, demonstrating promising potential as a precise regenerative platform based on the combination of stem cell recruitment and electrical stimulation-induced differentiation.

To improve cross-study comparison and highlight representative application strategies, Table 2 summarizes key piezoelectric material platforms reported for cartilage and osteochondral regeneration, including their fabrication methods, electrical outputs, biological outcomes, and proposed mechanisms of action.

As summarized in Table 2, current cartilage-related piezoelectric platforms have evolved from simple electrically responsive materials toward multifunctional constructs integrating structural guidance, bioactive regulation, and controllable electromechanical stimulation.

### 6.3. Summary and Perspectives

In summary, the application of piezoelectric materials in cartilage regeneration has gradually evolved from single-material electrical responsiveness toward integrated strategies that combine material design, structural engineering, and mechanistic regulation. Scaffold-based systems, injectable hydrogels, and responsive microsphere platforms each offer distinct advantages for reconstructing the electrical and biological microenvironment required for cartilage repair. Future research should focus on improving the precision of electrical output, controllability of biodegradation, and long-term tissue integration, while also strengthening mechanistic understanding and translational evaluation to facilitate eventual clinical application.

## 7. Conclusions and Future Perspectives

In recent years, piezoelectric materials have moved beyond their traditional roles in functional devices and emerged as promising bioactive platforms for bone and cartilage tissue engineering. Owing to their unique mechano-electrical coupling capability, these materials can convert physiological mechanical stimulation into localized electrical cues, thereby partially mimicking the endogenous electroactive microenvironment of musculoskeletal tissues. Through this property, piezoelectric materials have shown considerable potential in regulating cellular behavior, modulating the repair microenvironment, and promoting tissue regeneration.

Despite this encouraging progress, the clinical translation of piezoelectric biomaterials remains limited, and only a small number of electroactive systems have progressed toward real clinical application [59]. Several key barriers continue to hinder this transition. First, the physiological compatibility of piezoelectric stimulation parameters remains insufficiently defined, as different tissues, cell types, and pathological conditions may respond to distinct electrical amplitudes, frequencies, and temporal profiles. Second, it remains difficult to simultaneously achieve appropriate biodegradability, long-term electrical stability, structural integrity, and biological safety within a single material platform. Third, the biological effects of piezoelectric stimulation are mediated through complex and interconnected signaling networks, yet the underlying mechanisms are still incompletely understood, which limits the rational optimization of material design. In addition, the lack of standardized evaluation criteria for piezoelectric output, in vitro bioactivity, and in vivo regenerative performance makes it difficult to directly compare results across studies. Challenges related to scalable manufacturing, sterilization compatibility, reproducibility, long-term implantation safety, and regulatory approval further complicate clinical translation.

Future research should therefore focus on several major directions. First, it is necessary to develop multifunctional material systems that better balance electrical performance, flexibility, biodegradability, and biosafety, particularly through composite and hybrid design strategies suitable for complex osteochondral environments. Second, more precise and intelligent responsive platforms should be established to dynamically match the mechanical loading conditions and biological requirements of target tissues, thereby enabling better spatiotemporal control of piezoelectric stimulation. Third, deeper mechanistic investigations combining transcriptomics, proteomics, and other multi-omics approaches are needed to clarify how piezoelectric cues regulate tissue-specific repair pathways. Fourth, standardized preclinical evaluation frameworks should be established to improve cross-study comparability and accelerate translation from laboratory research to clinically relevant validation. Finally, advanced manufacturing methods, including 3D printing, microfluidics, and other customizable fabrication strategies, may further facilitate the development of structurally optimized and application-oriented piezoelectric regenerative constructs.

In summary, piezoelectric biomaterials represent a highly promising class of intelligent materials for bone and cartilage tissue engineering. Their future development will likely depend on the integration of materials science, bioelectrics, mechanobiology, regenerative immunology, and translational medicine. With continued advances in mechanistic understanding, material optimization, and standardized validation, piezoelectric materials may provide new opportunities for personalized and clinically effective repair strategies for complex musculoskeletal tissue injuries.

## Figures and Tables

**Table 1:** Representative piezoelectric coefficients of natural biological structures reported at different hierarchical and measurement scales.

Biological Structure/System	Main Structural Basis	Representative Piezoelectric Coefficient	Coefficient Type	Unit	Notes	Ref.
Amino acid crystals	Non-centrosymmetric amino acid crystal packing	0.5–176	various (often shear-dominant)	pC·N^−1^	Broad range reported across amino acid systems; high values are often measured/predicted in shear modes rather than longitudinal modes	[16,17,18]
Glycine crystal	Non-centrosymmetric β-phase glycine crystal	178 ± 11	d16	pC·N^−1^	Representative high-performance amino acid crystal; value reported for shear mode	[17,18]
Diphenylalanine (FF) nanotube	Ordered peptide self-assembly via hydrogen bonding and π–π stacking	35	d15	pC·N^−1^	Representative peptide-based biopiezoelectric system	[26]
Diphenylalanine (FF) vertical microrod array	Highly aligned FF microrod assembly	17.9	d33	pC·N^−1^	Useful as a longitudinal-mode reference for FF-based assemblies	[26]
Bone (human tibia)	Type I collagen-rich mineralized ECM	7.66–9.72	d33	pC·N^−1^	Representative tissue-scale value from human tibia	[28]
Bone collagen fibrils	Periodically organized type I collagen fibrils	0.2–2.8	general reported range	pC·N^−1^	Frequently cited range for collagen-based biological piezoelectricity; useful when discussing collagen as the structural source of tissue piezoelectricity	[30]
Cartilage	Type II collagen-rich ECM	0.2–0.7	d33	pC·N^−1^	Commonly cited range for cartilage tissue; reflects type II collagen contribution	[32]
Type II collagen fibrils	Triple-helical type II collagen	~20–32% of type I collagen d15	d15 (relative)	-	Often reported as lower than type I collagen rather than as a single absolute tissue value	[32]
Cornea (human)	Highly oriented lamellar collagen fibrils	~200/−660/−2250	anisotropic d-coefficients	pC·N^−1^	Horizontal/vertical/diagonal d15, respectively; strongly anisotropic and hydration-dependent	[35]
Sclera (human)	More disordered collagen organization	7–23 (circumferential), 6–8 (anterior–posterior)	anisotropic d-coefficients	pC·N^−1^	Often lower than cornea because of less ordered collagen alignment	[36]
Skin/dermal collagen	Collagen network	0.05–0.1	d14	pC·N^−1^	Shear piezoelectricity mainly attributed to dermal collagen	[29]
Skin epidermis (human)	Keratin-associated fibular structures	0.01–0.03	d14	pC·N^−1^	Lower than dermis and stratum corneum	[54]
Keratin-rich outer epidermal layer	Keratin-rich outer skin layer	0.1–0.2	d14	pC·N^−1^	Highest among separated dry skin layers in that dataset	[12,13,54]
Live human epidermis	Keratin-associated structures in living epidermis	0.03–0.28	d33	pC·N^−1^	Living tissue measurement; values higher than dry epidermis in some reports	[54]
Lysozyme crystal (monoclinic)	Monoclinic protein crystal, space group P2_1_	0.94	piezoelectric coefficient	pC·N^−1^	Measured along the [001] direction	[53]
Lysozyme crystal (tetragonal)	Tetragonal protein crystal, space group P4_3_2_1_2	3.16	piezoelectric coefficient	pC·N^−1^	Higher than monoclinic form due to stronger symmetry breaking	[53]
Outer hair cell Prestin system	Prestin-mediated electromechanical coupling in OHC lateral membrane	20	equivalent piezoelectric coefficient	μC·N^−1^	Exceptionally large equivalent coefficient; not directly comparable to standard bulk d33 tissue values	[49]

Note: Reported piezoelectric coefficients were collected from representative studies cited in this review. Direct comparison across biological systems should be made with caution because the measured values may vary depending on hydration state, crystallographic orientation, testing mode, sample preparation, and length scale.

**Table 2:** Summary of representative piezoelectric platforms for cartilage and osteochondral regeneration.

Reference	Material/Platform	Output/Activation	Key Biological Outcomes	Proposed Mechanism
Liu et al. [132]	dECM/Gel-PC bilayer scaffold	Self-generated output (−20 mV) under mechanical stimulation	Promoted region-specific differentiation; SOX9/COL2A1 upregulated in cartilage region; Runx2/ALP upregulated in bone region; supported osteochondral interface regeneration	Spatially distributed electrical cues induced tissue-specific lineage regulation
Xie et al. [133]	PLLA/BaTiO_3_ scaffold + collagen/FGF-18	Piezoelectric charge under loading; sustained FGF-18 release	Enhanced proliferation, ECM secretion, and type II collagen expression; improved cartilage repair in vivo	Piezoelectric stimulation synergized with biofactor release
Jacob et al. [134]	PHBV/BaTiO_3_ electrospun scaffold	d33 = 1.4 pC·N^−1^; poled/unpoled self-powered response	Promoted adhesion, proliferation, and COL2A1 expression; showed ECM similarity to native cartilage	Mimicked physiological piezoelectric microenvironment
Vinikoor et al. [135]	PLLA short-fiber/collagen hydrogel	Weak electrical signals under ultrasound	Promoted ADSC migration and chondrogenesis; upregulated SOX9, COL2A1, and ACAN; improved hyaline cartilage-like repair	Ultrasound-triggered piezoelectric stimulation enhanced endogenous TGF-β1—mediated repair
Han et al. [136]	Piezo@CR MPs (HAMA + BT + E7)	Max voltage ~451 mV produced under ultrasound	Increased ACAN, COL2A1, SOX9, and GAG synthesis; improved cartilage quality and functional recovery	Ca^2+^ influx and p38 MAPK activation, combined with stem cell recruitment

Note: Because material composition, activation mode, experimental model, and evaluation criteria vary substantially across studies, the reported outputs and biological outcomes should be interpreted within their specific experimental contexts.

## Data Availability

No new data were created or analyzed in this study. Data sharing is not applicable to this article.
